# Durability of Integrase STrand Inhibitor (InSTI)-based regimen in geriatric people living with HIV in the GEPPO cohort

**DOI:** 10.1371/journal.pone.0258533

**Published:** 2021-10-13

**Authors:** Emanuele Focà, Andrea Calcagno, Stefano Calza, Stefano Renzetti, Annacarla Chiesa, Matteo Siano, Giuseppe De Socio, Stefania Piconi, Giancarlo Orofino, Giordano Madeddu, Anna Maria Cattelan, Silvia Nozza, Micol Ferrara, Jovana Milic, Benedetto Maurizio Celesia, Francesco Castelli, Giovanni Guaraldi

**Affiliations:** 1 Department of Infectious and Tropical Diseases, University of Brescia and ASST Spedali Civili Hospital, Brescia, Italy; 2 Unit of Infectious Diseases, Department of Medical Sciences, University of Torino, Torino, Italy; 3 Unit of Biostatistics and Bioinformatics, Department of Molecular and Translational Medicine, University of Brescia, Brescia, Italy; 4 BDbiomed lab, University of Brescia, Brescia, Italy; 5 3rd Division of Infectious Diseases, University of Milano, L. Sacco Hospital, Milano, Italy; 6 Department of Infectious Diseases, Azienda Ospedaliero-Universitaria di Perugia, Perugia, Italy; 7 1st Division of Infectious Diseases Unit, University of Milano, L. Sacco Hospital, Milano, Italy; 8 Unit of Infectious Diseases, ’Divisione A’, Amedeo di Savoia Hospital, ASLTO2, Torino, Italy; 9 Unit of Infectious and Tropical Diseases, Department of Medical, Surgical and Experimental Sciences, University of Sassari, Sassari, Italy; 10 Unit of Infectious Diseases, Department of Internal Medicine, Azienda Ospedaliero-Universitaria di Padova, Padova, Italy; 11 Department of Infectious Diseases, San Raffaele Scientific Institute, Milano, Italy; 12 Department of Mother, Child and Adult Medicine and Surgical Science, Infectious Disease Clinic, University of Modena and Reggio Emilia, Modena, Italy; 13 Division of Infectious Diseases, Department of Clinical and Molecular Biomedicine, University of Catania, ARNAS Garibaldi, Catania, Italy; Rush University, UNITED STATES

## Abstract

**Objective:**

To investigate the durability of the first integrase inhibitor-based regimen in a HIV geriatric multicentric prospective cohort and to explore the reasons of regimen discontinuation.

**Design:**

This is an analysis conducted on the Geriatric Patients Living with HIV/AIDS (GEPPO) cohort, an Italian prospective observational multicentre cohort of people living with HIV with 65 years of age or more.

**Methods:**

The analysis was performed using R (version 4.0.2). The tests performed were two sided assuming a 5% significance level (Kruskal-Wallis test, Chi-squared test, log-rank test and a Cox Proportional Hazard model). The proportion of participants discontinuing the three regimens was displayed using cumulative curves.

**Results:**

Among 1531 patients enrolled between 2017 and 2019 in the GEPPO cohort, we included 822 participants in this analysis. At baseline, median age was 69.8, the immunovirological profile good, multimorbidity was present in 42.3% of participants, while 27.4% were on polypharmacy. Overall, 483, 243 and 96 participants received DTG, RAL and EVG/c respectively as first InSTI. At the end of the follow up 6.4%, 21.1% and 22.9% participants discontinued DTG, RAL and EVG/c respectively. Using a log-rank test, EVG showed a significantly lower durability than DTG (p<0.001) or RAL (p 0.05) or both, DTG and RAL (p<0.001). Among participants who discontinued their regimen we found 0 virological failure and 56.7% simplification/deprescription.

**Conclusions:**

The three integrase inhibitors considered showed a good durability and no virological failures in geriatric patients such as those enrolled in the GEPPO cohort when used in a two or three drug regimen.

## Introduction

People living with HIV (PLWH) are aging, mainly for the longer life expectancy under effective combined antiretroviral therapy (cART) and the increasing age of newly diagnosed patients [1, 2]. Geriatric populations (conventionally considered as people with 65 or more years of age) present a peculiar pattern of health necessity. An integrated management is therefore needed to guarantee a healthy aging because PLWH have a higher risk of age-associated events compared with the same non-HIV age cohorts [3]. For this reason a specific focus on elderly PLWH is being implemented in several guidelines [4, 5].

Multimorbidity (i.e. the presence of at least 3 co-morbidities) is often present in elderly PLWH and it is reported along with polypharmacy (i.e. the concomitant intake of at least 5 non-antiretroviral medications): both conditions have been associated with worse outcomes [2, 6].

Moreover, there is a lack of randomized controlled trials (RCTs) designed properly to investigate efficacy and toxicity of antiretrovirals in geriatric patients living with HIV. Recently some sub-studies of RCTs including a ≥65 years old sub-group analysis were presented and only one non-randomized single arm clinical trial showing good tolerability, safety profile and immunovirological response with InSTI-based cART in geriatric PLWH both for cART experienced and naïve patients [7, 8].

One of the methods that may empirically show the tolerability of a drug regimen is its durability. Durability, defined as the time to regimen modification, depends on major outcomes (e.g. allergic reactions, virological failure or drug-drug interactions) as well as on more subtle factors such as mild side effects, the impact on quality of life, polypharmacy, comorbidities and patients’ and physicians’ choice [9]. Integrase Strand Transfer Inhibitor (InSTI) containing regimens are the preferred treatment in naïve patients by several international guidelines: the class includes raltegravir (RAL), elvitegravir/cobicistat (EVG/c), dolutegravir (DTG) and bictegravir (BIC, only recently added to the armamentarium with very limited real-life data) [4]. To date, no studies investigated the durability of InSTIs in geriatric PLWH. Therefore, in order to contribute filling the gap in current knowledge, we aimed to explore the durability of InSTI-based regimens in a geriatric cohort of PLWH.

## Methods

Participants were prospectively recruited between 2017 and 2019 from the Geriatric Patients Living with HIV/AIDS (GEPPO) cohort, an Italian prospective observational multicentre study characterising multimorbidity and geriatric syndromes in PLWH aged ≥65 years with a special focus on cART prescription. Participants are followed according to clinical practice and data about geriatric outcomes are recorded once a year. Regarding this study, inclusion criteria was the initiation of first InSTI-based regimen after 2017. InSTIs included in the analysis were DTG, RAL and EVG/c. Moreover, we explored the presence of multi-morbidity and polypharmacy: those conditions have been previously defined and described in the GEPPO cohort by *Nozza et al* [10].

Study primary endpoint was InSTI durability defined as time to discontinuation of first InSTI regimen; we also analyzed the reason for cART discontinuation, categorized as: 1) virological failure, 2) simplification/deprescription, 3) others (including toxicity).

### Statistical analysis

Continuous variables were described using median and interquartile range (IQR) while categorical variables were described with absolute frequencies and percentages. Comparisons of continuous variables among InSTI regimes were performed using Kruskal-Wallis test while Chi-squared test was used for categorical variables. The difference of InSTI discontinuation, considered as the first regimen shift and modelled as a time-to-event variable, was tested through a log-rank test and a Cox Proportional Hazard model adjusting by age, sex, multimorbidity (defined as the presence of at least 3 comorbidities) and the use of abacavir. The same analysis was conducted for the subgroup on InSTI dual regimen. Median survival is defined as the time needed for half of the participants in each regimen to discontinue the treatment. The proportion of participants discontinuing the three regimens was displayed using cumulative curves. All the test performed were two sided assuming a 5% significance level. All analyses were performed using R (version 4.0.2).

### Ethics statement

This study belongs to the GEPPO project. This project obtained approval by the Research Ethics Board of each individual institute belonging to the GEPPO cohort. The Research Ethics Board of the coordinating centre is the “*Comitato Etico di Brescia*”. All data were fully anonymized before the statistical analysis was performed. Written informed consent was asked at the first useful access.

## Results

Among 1531 patients aged ≥65 years followed in the GEPPO cohort, we included 822 participants in this analysis. At baseline (defined as the time of inclusion in the analysis) median age was 69.8 (IQR 7.4). Among study participants, 666 (81%) were males, the median CD4+ T-cell count at baseline was 618 cell/μl (IQR 25–75: 437.5–814.0), and 95.2% had HIV RNA <50 copies/ml. Multimorbidity was present in 356 (43.3%) participants, while 225 (27.4%) were on polypharmacy (all summary statistics are reported in Table 1).

**Table 1:** Overall and divided by first InSTI groups summary statistics of the variables considered in the study: Median and interquartile range are shown for continuous variables while counts and percentages are considered for categorical variables. Kruskal-Wallis test and Chi-squared test were used for continuous and categorical variables respectively.

	DTG (N = 483)	EVG (N = 96)	RAL (N = 243)	Total (N = 822)	p value
**Age (years)**					0.020
Median (Q1, Q3)	69.8 (67.0, 74.4)	69.0 (66.3, 71.6)	70.6 (67.3, 75.6)	69.8 (67.0, 74.4)	
**Sex**					0.645
Female	88 (18.2%)	15 (15.6%)	52 (21.4%)	155 (18.9%)	
Male	394 (81.6%)	81 (84.4%)	191 (78.6%)	666 (81.0%)	
Transgender	1 (0.2%)	0 (0.0%)	0 (0.0%)	1 (0.1%)	
**Multi-Morbidities (at least 3 comorbidities)**					0.014
No	284 (58.8%)	62 (64.6%)	120 (49.4%)	466 (56.7%)	
Yes	199 (41.2%)	34 (35.4%)	123 (50.6%)	356 (43.3%)	
**Polypharmacy (>5 medications)**					< 0.001
• No	367 (76.0%)	78 (81.2%)	152 (62.6%)	597 (72.6%)	
• Yes	116 (24.0%)	18 (18.8%)	91 (37.4%)	225 (27.4%)	
**HAART duration (years)**					0.001
Median (Q1, Q3)	16.1 (10.3, 20.4)	13.5 (8.5, 19.9)	18.3 (12.0, 21.1)	16.4 (10.2, 20.7)	
**CD4**					0.401
Median (Q1, Q3)	610.0 (443.0, 826.0)	651.0 (435.5, 853.8)	618.0 (440.8, 733.8)	618.0 (437.5, 814.0)	
**HIV suppressed (not detectable or RNA < 50)**					0.125
• No	13 (4.6%)	5 (10.4%)	3 (2.9%)	21 (4.8%)	
• Yes	268 (95.4%)	43 (89.6%)	102 (97.1%)	413 (95.2%)	
**DUAL (InSTI + other)**					< 0.001
• No	424 (87.8%)	NA	189 (77.8%)	709 (86.3%)	
• Yes	59 (12.2%)	NA	54 (22.2%)	113 (13.7%)	
**DUAL (InSTI + Lamivudine)**					< 0.001
• No	394 (81.6%)	NA	242 (99.6%)	732 (89.1%)	
• Yes	89 (18.4%)	NA	1 (0.4%)	90 (10.9%)	
**DUAL (InSTI + Rilpivirine)**					< 0.001
• No	436 (90.3%)	NA	240 (98.8%)	772 (93.9%)	
• Yes	47 (9.7%)	NA	3 (1.2%)	50 (6.1%)	
**TRIPLE (InSTI + 2 others)**					< 0.001
• No	207 (42.9%)	5 (5.2%)	84 (34.6%)	296 (36.0%)	
• Yes	276 (57.1%)	91 (94.8%)	159 (65.4%)	526 (64.0%)	
**MEGA (InSTI + at least 4 others)—molecules**					< 0.001
• No	471 (97.5%)	91 (94.8%)	217 (89.3%)	779 (94.8%)	
• Yes	12 (2.5%)	5 (5.2%)	26 (10.7%)	43 (5.2%)	
**Abacavir**					< 0.001
• No	325 (67.3%)	96 (100.0%)	216 (88.9%)	637 (77.5%)	
• Yes	158 (32.7%)	0 (0.0%)	27 (11.1%)	185 (22.5%)	
**Single Tablet Regimen**					< 0.001
• No	330 (68.3%)	5 (5.2%)	243 (100.0%)	578 (70.3%)	
• Yes	153 (31.7%)	91 (94.8%)	0 (0.0%)	244 (29.7%)	

NA: not applicable.

Overall, 483, 243 and 96 participants received DTG, RAL and EVG/c respectively as first InSTI. At the baseline, participants were on cART from a median (IQR 25–75) of 16.1 (10.3–20.4), 18.3 (12.0–21.1) and 13.5 (8.5–19.9) years in the DTG, RAL and EVG/c group respectively, with significant differences among the three InSTIs (Kruskal-Wallis test, p = 0.001).

**Fig 1:**
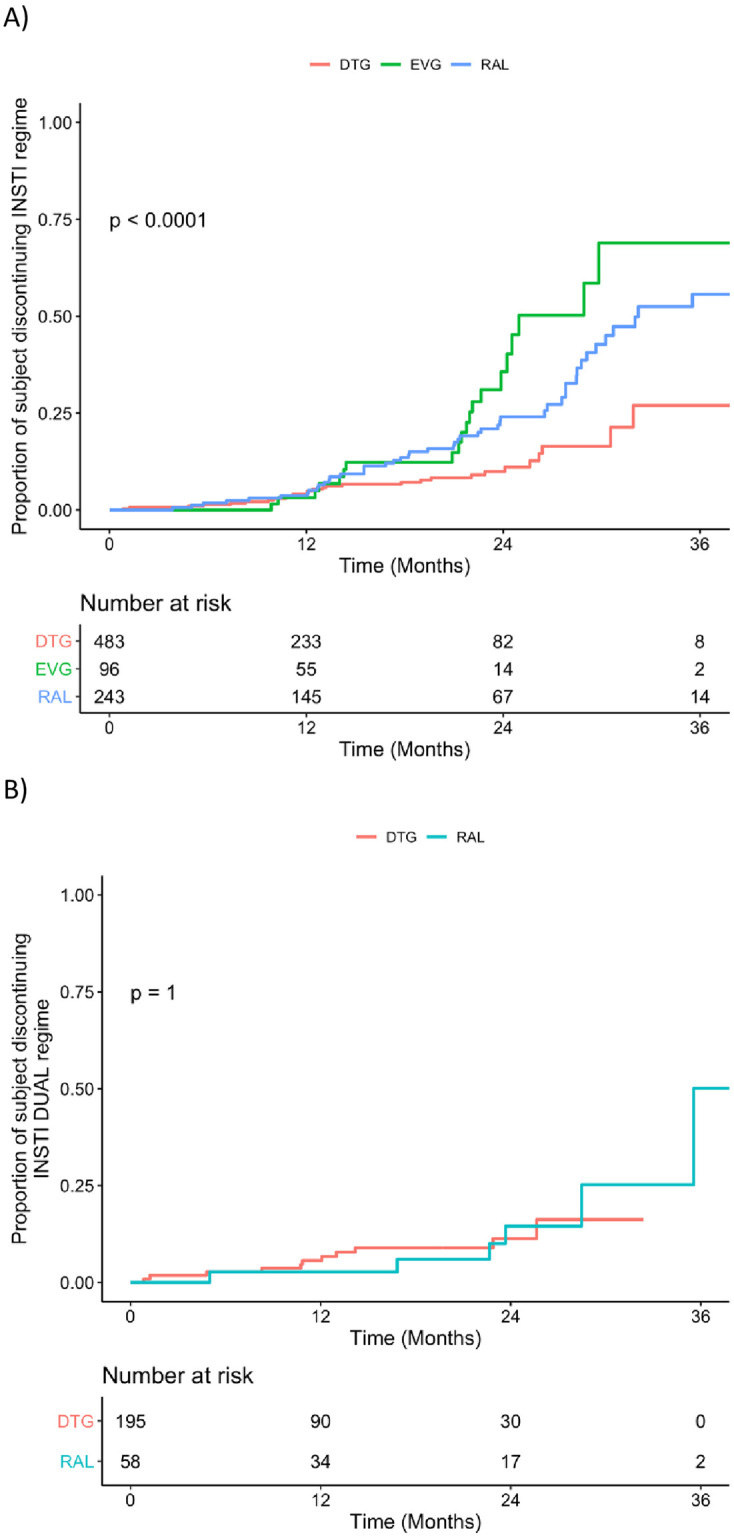
Curves showing the proportion of subjects discontinuing the InSTI regime. Comparison performed using log-rank test for the whole cohort—p < 0.0001—(A), and the subgroup of patients undergoing a dual drug regimen (B).

The median follow-up time, computed with KM estimator, was 12.7 months. At the end of the follow up 29 (6.0%), 52 (21.4%) and 22 (22.9%) participants discontinued DTG, RAL and EVG/c respectively. The median discontinuation time calculated as the time required to reach a drug discontinuation of 50% was 29 months for EVG and 36 months for RAL while DTG did not reach 50% discontinuation (after 36 months the proportion of participants who discontinued DTG was 23%). Using a log-rank test, EVG showed a significantly lower durability than DTG (p<0.001) or RAL (p = 0.048) or both DTG and RAL (p<0.001). The curves showing the proportion of participants discontinuing InSTI regimen are shown in Fig 1A. When adjusting for age, sex, multimorbidity and abacavir usage we were still able to find a significant difference between DTG and EVG but not between RAL and EVG (p = 0.002 and p = 0.111 respectively, Table 2).

**Table 2:** Results obtained applying a Cox regression to test the difference in time to discontinuation among InSTI group adjusting for age, sex, multimorbidity and abacavir use.

*Predictors*	*Estimates*	*CI*	*p*
InSTI: DTG vs EVG	0.24	0.09–0.59	**0.002**
InSTI: RAL vs EVG	0.43	0.15–1.21	0.111
Age	1.00	0.97–1.04	0.920
Sex: Male vs Female	1.11	0.68–1.82	0.663
Multimorbidity: Yes vs No	0.67	0.44–1.02	0.064
Abacavir: Yes vs No	0.85	0.41–1.75	0.658
STR: Yes vs No	0.63	0.25–1.57	0.319

An intriguing treatment option recently implemented thanks to several RCTs, are two drug regimens (2DR) InSTI-based. Analyzing this treatment choice, we found that overall 526 (64.0%) of the participants received a standard triple therapy while 195 (40.4%) received DTG-based 2DR and 58 RAL-based 2DR. Among those receiving DTG-based 2DR, for 89 (45.6%) participant the second drug was lamivudine (3TC), for 47 (24.1%) rilpivirine (RPV), and for 59 (30.3%) other agents. Durability of InSTI-based 2DR is shown in Fig 1B (EVG/c is not included because it is available in 3DR regimen only).

Among participants who discontinued their regimen (103) we found 0 (0%) virological failure, 59 (56.7%) simplification/deprescription, 11 (25.9%) other reasons, including 4 adverse events and 1 participant’s preference. For the remaining 33 participants, data regarding the reason of InSTI discontinuation are missing, although among them 4 participants changed molecules in the InSTI class.

## Discussion

This longitudinal study in geriatric PLWH demonstrated a good durability of InSTI-based regimens analysed with a particularly good performance of DTG-based regimens (regardless of accompanying drugs).

Geriatric Patients Living with HIV/AIDS (GEPPO) is a prospective observational cohort including consecutive HIV-positive geriatric patients in care at 11 HIV clinics all over Italy compared with a HIV-negative control group. The aim is monitoring the health status of geriatric PLWH as well as study how the common geriatric care model applies to PLWH [11].

The 822 participants at GEPPO cohort also enrolled in this study were long standing cART experienced with a median history of more than 10 years of antiretroviral drugs and presenting a good immunovirological profile. Aging is usually associated with a higher tendency to treatment adherence [2] which may explain their good immunovirological profile. However, a recent report from GEPPO Cohort showed that only the 31% experienced a normalization of immunological profile with a significant difference between genders [12, 13].

As expected, in a cohort of people with a median age of 70 years, multimorbidity and polypharmacy were common findings (42 and 27%, respectively). It is well known that these two factors should be carefully considered when choosing an ART regimen: if virological suppression is achieved, NRTI-sparing, unboosted regimens are emerging as intriguing treatment options as well as the possibility to simplify to dual therapy should be considered [2]. Notably, tenofovir alafenamide should be preferred to tenofovir diproxil fumarate due to lower toxicity on bone mineral density and renal function [2]. Moreover, in our cohort Guaraldi *et al*. did not observe a weight gain in participant switching from tenofovir diproxil fumarate to tenofovir alafenamide nor from a InSTI-free cART to a DTG-based regimen [14].

The emerging interest on DTG-based 2DR has been extensively investigated in RCTs when DTG was combined/co-formulated with RPV or 3TC [15]. These regimens, beyond virological durability harbor pharmacokinetic benefits of low burden of DDI and single tablet regimen (STR) convenience.

These data are confirmed in our cohort. None of the participants discontinued due to virological failure, while 56.7% for simplification/deprescription, and 25.9% for toxicity. These data are in line with what found by *Eaton et al*. [9]: ART changes due to virological failure are less and less common in recent years while most switches are due to convenience and side effects. Moreover, the low rate of transmitted resistance-associated mutations to InSTIs and the high genetic barrier of this class contributes explaining this result [16].

These data may also be put in the perspective of the fourth 90% target recently suggested as outcome in the HIV treatment cascade. Switching ARV in geriatric PLWH is mainly driven by a proactive strategy to avoid polypharmacy in a de-prescribing approach, often applied in general geriatric population [17]. In this context, given the long durability of the prescribed regimen, this study contributes to generate new data regarding safety of both 3DR and 2DR InSTI based regimen in this particular population.

EVG and RAL also showed a good durability (29 and 36 months respectively), though they were less preferred in GEPPO cohort in comparison to DTG. A few hypotheses may be provided.

EVG is necessarily boosted with cobicistat which strongly inhibit CYP3A4 displaying a higher EVG risk of DDIs, producing concerns in the context of polypharmacy. RAL discontinuation may be due to high pill burden regardless of the once a day RAL formulation which has been recently released. To note RAL anyway showed a good durability, higher than EVG.

In geriatric PLWH deprescription options should be the standard of care. This process implies therapy review with the goal of medication reconciliation and medication prioritizations to prevent unnecessary drug prescriptions. In this context 2DR and 3DR unboosted STR regimens should be prioritised [18]. Recent data on STR bictegravir-based treatments in elderly participants showed excellent efficacy and tolerability: the randomized clinical setting support further real-life investigations [7].

Several limitations of our study should be acknowledged: missing data, short follow up, only one time-point per year, and the observational design, unable sometimes to evaluate the clinical decisions.

## Conclusion

Although the three InSTI considered showed some differences in this geriatric cohort of patients, all of them had a durability of more than two years with no virological failures. In geriatric patients such as those enrolled in the GEPPO cohort 2DR and 3DR unboosted STR regimens are favoured and display long term durability.

## Supporting information

S1 Data (XLSX)
